# Fibrin Glue Assisted Trans-Scleral Fixation of an Endocapsular Device for Sutureless Trans-Scleral Capsular Bag Fixation in Traumatic Subluxations: The Glued Endocapsular Ring/Segment

**Published:** 2013

**Authors:** Soosan Jacob, Amar Agarwal

**Affiliations:** Dr. Agarwal's Eye Hospital and Eye Research Centre; 19, Cathedral Road, Chennai- 600 086, India

**Keywords:** Subluxated cataracts, Subluxated IOL, Glued endocapsular ring, Fibrin Glue Assisted Trans-Scleral Fixation, Sutureless fixation

## Abstract

Subluxated cataracts secondary to blunt injury are a challenge to treat from the construction of rhexis to IOL insertion. For that reason, we designed a new glued endocapsular ring (ECR)/ segment (ECS) for subluxated cataracts and IOLs for sutureless fibrin glue assisted trans-scleral fixation of the capsular bag. The glued ECR/segment stabilizes the capsular bag intra and post-operatively, allowing for sutureless fibrin glue assisted trans-scleral fixation. The segment gives vertical, horizontal and rotational stability as well as forniceal expansion. The advantages to this approach include easier implementation; faster surgery; easy adjustability; sturdier scleral fixation; fewer chances of segment drop into vitreous and lack of suture-related complications. Our hypothesis is that a glued versus sutured ECR/ECS will be more viable and stable on the sclera in the long term. Less pseudophakodonesis will also lead to a more stable capsule-bag complex and reduce the risk of posterior segment complications such as retinal detachment and cystoid macular edema. The nature of the device also makes its removal, if required, much more straightforward than the sutured rings/segments. This device can be used in patients with subluxated cataracts, colobomatous lens or subluxated IOLs.

## INTRODUCTION

Ocular trauma is a major cause of ocular morbidity and can result in various types of injuries to the eye. These include penetrating injuries and blunt injuries to the eye [1,2] ranging from corneal tears to avulsion of the optic nerve. These are commonly seen in the younger age group and can culminate in significant morbidity. Though both penetrating injuries and blunt injuries can manifest as traumatic subluxation of the lens, the presentation can be very different. Subluxation in penetrating ocular trauma is usually associated with a corneal, scleral or corneo-scleral tear, uveal tissue prolapse, and frequently with anterior, and occasionally posterior lens capsule tear[3]. This generally results in complicated surgery including the need to repair the integrity of the globe as well as cataract extraction with primary or secondary IOL implantation.

Subluxations associated with blunt injuries generally have an intact anterior and posterior capsule. The extent of subluxation depends on the extent of zonulodialysis resulting from the concussion injury and can lead to a decrease in vision. It may also be associated with the development of a post-traumatic rosette cataract which can lead to a further decrease in vision. The classical manners in which such subluxations are dealt with depend on the extent of dialysis and the degree of nuclear sclerosis [4]. For small subluxations, an endocapsular ring may suffice [5-11]. But for subluxations affecting more than three to four clock hours, scleral fixation of the capsular bag and its contents is required [12-15]. This is traditionally done with sutured endocapsular segments and rings [12-14]. The need for sutures has the inherent disadvantage of long term viability and can lead to problems associated with strength and durability.

## HYPOTHESIS

We hypothesize that a device that can expand and stabilize the endocapsular bag without the use of sutures will not only have better stability in the long term but will also provide better stability intra-operatively, i.e., be able to withstand the stress forces that are exerted during surgery. For this we designed a new intra-ocular device the glued endocapsular ring/segment to provide fibrin glue assisted sutureless trans-scleral fixation of the capsular bag and its contents [16]. This device was designed by one of the authors (SJ).

## DISCUSSION

Subluxated cataracts can be a challenge in surgery. A lax bag does not offer sufficient resistance to all the manoeuvres that are required for a successful cataract surgery. This can lead to difficulties in every step, right from creating the rhexis to implanting the IOL [5]. Subluxations smaller than 3-4 clock hours can be managed by capsular tension rings [6-11] but larger subluxations require scleral fixation of the capsular bag [12-15]. For such larger subluxations, once the rhexis is created, one option is to perform a hydrodissection, implant the conventional sutured endocapsular ring or segment and fix it to the scleral wall by means of a suture [12-15]. The rest of the cataract surgery is then performed followed by lens implantation. The second option is to perform a rhexis, use capsule hooks [17-20] to stabilize the bag and then proceed with cataract surgery. This necessitates having to make multiple paracenteses wounds to insert the capsule hooks which then need to be removed and replaced with a sutured endocapsular ring/segment at the end of phacoemulsification. 

The disadvantages of the first option include the lack of stability provided by sutured fixation. Sutures that have been used include 10-0 and more recently 9-0 prolene or GoreTex. The suture acts as a link between the endocapsular ring at one point and the sclera at the other point. As it is a single point fixation at both ends, it leads to an increased possibility of pseudophacodonesis as well as rotational movements of the device and therefore the capsular bag. Sutures also do not provide the sufficient amount of vertical stability that is required for in the bag manoeuvres such as chopping and cracking. Frequently, the entire bag is moving while attempting in the bag manoeuvres. The intra-operative disadvantage of the second option involves an increased number of incisions for inserting capsule hooks. More incisions can potentially increase the risk of post-operative complications such as wound leak, shallow anterior chamber, endophthalmitis, etc. Once the hooks are replaced by the sutured endocapsular ring/ segment prior to IOL insertion, the disadvantages associated with sutured capsular bag fixation are present.

In addition to these disadvantages, sutured segments/rings also have the inherent disadvantage of requiring more complicated intra-ocular manoeuvring for implanting the endocapsular ring. The suture needs to be tied to the endocapsular segment/ring and then requires multiple passes of long and thin needles across the anterior chamber. This makes the procedure more complicated and time consuming. Also, once the sutures are brought out under the lamellar scleral flap, they are tied down to keep the ring in place. Any miscalculation in the tension with which the knot is tied can result in either an insufficient centring of the capsular bag or a lax, dangling bag which can further increase the occurrence of pseudophacodonesis. Should this happen, it is necessary to cut the knot and repeat the entire procedure.

**Fig 1:**
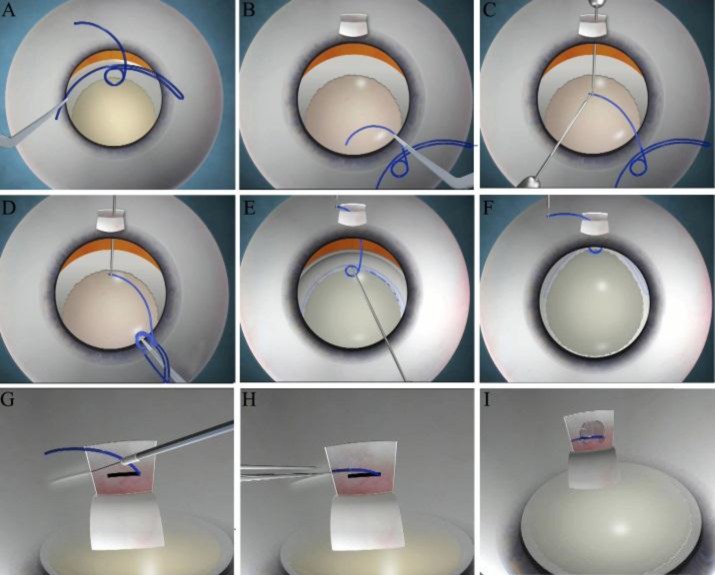
A: The design of the glued endocapsular hemi-ring is seen as well as its positioning in the direction of dialysis. B: a lamellar scleral flap and sclerotomy are created in the area of dialysis. The haptic of the device is introduced into the anterior chamber. C: The haptic is caught by an end-gripping microforceps introduced through the sclerotomy. D: The rest of the glued endocapsular hemi-ring is flexed in using a singlehanded fish-tailing technique. E: The circular scrolls engage the capsulorhexis rim. F: The capsular bag is centered. G: A scleral tunnel is created at the edge of the scleral flap with a 26-gauge needle. H: The haptic is tucked into the tunnel. I: Fibrin glue is applied and the flap is sealed down over the haptic.

Reprinted from Journal of Cataract and Refractive Surgery 38(2); Jacob S, Agarwal A et al. Glued endocapsular hemi-ring segment for fibrin glue–assisted sutureless transscleral fixation of the capsular bag in subluxated cataracts and intraocular lenses. PP: 193-201 Copyright 2012 with permission from Elsevier.

The glued endocapsular ring is designed to have an intra-capsular forniceal part which consists of two arms, a Malyugin ring-like scrolled engaging mechanism [21,22] which engages the rim of the rhexis and a haptic which is exteriorized through a sclerotomy under a scleral flap. The haptic gets tucked into an intra-scleral coat hanger shaped Scharioth tuck [23,24]. The scleral flap is glued down with fibrin glue as in a glued IOL technique [25-27](Fig 1). The advantage to this device is that part of it is exteriorized out through a sclerotomy and tucked trans-sclerally along a significant part of its length. As it is a part of the device itself that is exteriorized and tucked, we hypothesize that it leads to greater rotational and vertical stability both intra- and post-operatively. The tight fit within the scleral tunnel does not allow horizontal, vertical or rotational movements of the ring and thereby the capsular bag. Elimination of single point fixation at both ends- - at the ring and at the scleral point of attachment - gives the glued endocapsular ring an inherent stability that is not seen with sutured rings/ segments. This procedure, viewed in its entirety, leads to decreased pseudophacodonesis and concurrently, a decreased incidence of posterior segment complications. 

**Fig 2:**
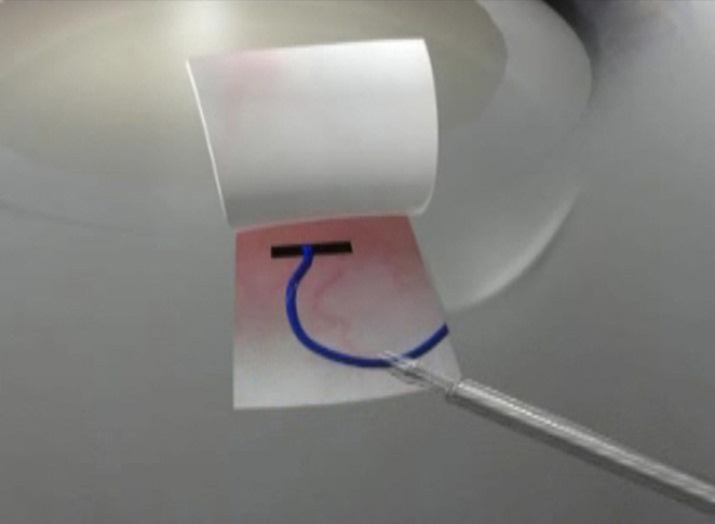
An intra-scleral coat hanger shaped Scharioth tuck is preferred to anchor the haptic to the sclera.

As the gauge of the glued endocapsular ring is designed to be the same as that of the IOL haptic, it also provides more robust fixation in the intra- and post-operative period. This provides good stability for performing in the bag manoeuvres such as nucleus chopping, etc. Long term problems that are associated with sutured fixation such as suture degradation, erosion and knot slippage are eliminated [28-34]. The coat hanger type of intra-scleral tunnel and trans-scleral fixation that is used effectively anchors the ring onto the sclera without any chance of slippage (Fig 2). Since it is constructed of IOL haptic material, its long term biocompatibility both within the eye as well as trans-sclerally is also known [23-27]. The Malyugin ring-like scrolled engaging mechanism that is used for engaging the rhexis is atraumatic. 

Intra-operative advantages of this approach include ease of surgery. The trans-chamber passage of long, thin needles is no longer required, so this surgery has become a relatively straight-forward procedure. Also, there is less operating time needed. Elimination of sutures and knots makes intra-operative adjustability very easy. The length of the externalised haptic is cut to the desired length and the degree of tuck of the haptic within the intra-scleral tunnel is adjusted till good centration is obtained. This process can again be repeated after IOL implantation so that the IOL is well centered on the visual axis. 

In any kind of scleral bag fixation - either sutured or sutureless - a decentered bag (due to a lack of centration of the sclera flap on the extent of dialysis) could occur regardless of the tension of the sutures or the degree of tuck of the externalised haptic. If this occurs, the scleral flap needs to be re-fashioned at the correct location again to ensure centration. With sutured segments/ rings, it then becomes necessary to perform the entire procedure all over again, from tying the knot onto the endocapsular ring/segment. But with the glued endocapsular ring/segment, it is only necessary to interiorise the haptic into the anterior chamber and then re-exteriorize it out again through a sclerotomy under the correctly placed new flap. 

It is also easy to remove the ring should the need arise as it is made of flexible material and can easily be pulled out. The use of fibrin glue to seal the flap makes the procedure much safer as the haptic is sealed hermetically under the flap with no portion of it exposed to the sub-conjunctival space [25-27].

In an IRB approved, prospective non-comparative case series, 5 eyes of 5 patients (three males, two females) with traumatic subluxated cataract (extent of subluxation ranging from more than 120 degrees to 180 degrees) underwent glued endocapsular ring implantation. The mean age was 49.8 ± 8.22 years. These included one inferior, one supero-nasal, one infero-nasal, and two superior subluxations. The device was easy to implant and provided adequate capsular support in all patients. It also allowed in-the-bag IOL implantation in all patients. BCVA in decimal equivalent improved from a mean of 0.316±0.123 pre-operatively to 0.893±0.153 at 7 months post-operative period. Pre-operative mean IOP by non contact tonometry was 14.6±4.34 and 7 months post-operative mean IOP was 12.8±3.03. Pre-operative specular count was 2837.8±214.811 and 7 months post-operatively, it was 2596.8±196.412. Central foveal thickness on macular OCT at 7 months post-operative period was 238.8±25.05.

## CONCLUSION:

The glued endocapsular ring/ segment enables sutureless trans-scleral fixation of the capsular bag and its contents. It provides good horizontal, vertical and rotational stability in both the intra-operative and post-operative period as well as capsular expansion. Glued ECR/ ECS as versus sutured segments/ rings have greater long term stability attained by the bag, are less complicated procedurally and are easier to adjust.
